# Concurrent agreement between an anthropometric model to predict thigh volume and dual-energy X-Ray absorptiometry assessment in female volleyball players aged 14-18 years

**DOI:** 10.1186/s12887-016-0730-7

**Published:** 2016-11-24

**Authors:** Óscar M. Tavares, João Valente-dos-Santos, João P. Duarte, Susana C. Póvoas, Luís A. Gobbo, Rômulo A. Fernandes, Daniel A. Marinho, José M. Casanova, Lauren B. Sherar, Daniel Courteix, Manuel J. Coelho-e-Silva

**Affiliations:** 1 UID/DTP/03213/2016, Faculty of Sport Sciences and Physical Education, University of Coimbra, Pavilhao III, 3040-156 Coimbra, Portugal; 2 Department of Medical Imaging and Radiation Therapy, School of Health and Technology, Instituto Politécnico de Coimbra, Coimbra, Portugal; 3 Faculty of Physical Education and Sport, Lusófona University of Humanities and Technologies, Lisbon, Portugal; 4 Portuguese Foundation for Science and Technology (SFRH/BPD/100470/2014), Lisbon, Portugal; 5 Research Centre in Sport and Physical Activity, Maia Institute of Higher Education, Maia, Portugal; 6 Laboratory of Investigation in Exercise (LIVE), Department of Physical Education, São Paulo State University (UNESP), São Paulo, Presidente Prudente Brazil; 7 Department of Sport Sciences, University of Beira Interior, Covilhã, Portugal; 8 Faculty of Medicine, University of Coimbra, Coimbra, Portugal; 9 School of Sport, Exercise and Health Sciences, Loughborough University, Loughborough, UK; 10 Laboratory of Metabolic Adaptations to Exercise in Physiological and Pathological Conditions, Clermont Auvergne University, Blaise Pascal University, Clermont-Ferrand, France; 11 School of Exercise Science, Faculty of Health, Australian Catholic University, Melbourne, VIC Australia; 12 Research Centre in Human Nutrition, Auvergne Clermont-Ferrand, France

**Keywords:** Adolescent female athletes, Body composition, Deming regression, PRESS

## Abstract

**Background:**

A variety of performance outputs are strongly determined by lower limbs volume and composition in children and adolescents. The current study aimed to examine the validity of thigh volume (TV) estimated by anthropometry in late adolescent female volleyball players. Dual-energy X-ray absorptiometry (DXA) measures were used as the reference method.

**Methods:**

Total and regional body composition was assessed with a Lunar DPX NT/Pro/MD+/Duo/Bravo scanner in a cross-sectional sample of 42 Portuguese female volleyball players aged 14–18 years (165.2 ± 0.9 cm; 61.1 ± 1.4 kg). TV was estimated with the reference method (TV-DXA) and with the anthropometric method (TV-ANTH). Agreement between procedures was assessed with Deming regression. The analysis also considered a calibration of the anthropometric approach.

**Results:**

The equation that best predicted TV-DXA was: -0.899 + 0.876 × log_10_ (body mass) + 0.113 × log_10_ (TV-ANTH). This new model (NM) was validated using the predicted residual sum of squares (PRESS) method (R^2^_PRESS_ = 0.838). Correlation between the reference method and the NM was 0.934 (95%CI: 0.880–0.964, S_y∙x_ = 0.325 L).

**Conclusions:**

A new and accurate anthropometric method to estimate TV in adolescent female volleyball players was obtained from the equation of Jones and Pearson alongside with adjustments for body mass.

**Electronic supplementary material:**

The online version of this article (doi:10.1186/s12887-016-0730-7) contains supplementary material, which is available to authorized users.

## Background

Human structure is commonly viewed as a hierarchy of organism, system, organ, tissue and cell [1, 2]. Consequently, body composition may be approached at different levels. Total body cannot be considered as a compositional level and the cellular level is of limited and specific interest. Taking these previous notes into account, the organizational system is often reduced into the *chemical* and the *anatomical* levels as proposed by Martin and Drinkwater [3].

Research dealing with body composition is often focused in the absolute quantity, percentage and distribution of body fat, in part, because of the apparent negative relationship of the fat component to health status. In the context of many sports, changing total and regional muscle component is a determinant factor in performance [4, 5] and the limited literature [6] suggests that total and appendicular lean soft tissue estimates have probably not received enough attention when compared to fat mass.

Lean soft tissue estimates derived from tissue-based models have been previously proposed [7–13]. A variety of fitness measures in youth were shown to be strongly determined by appendicular skeletal muscle mass [14–16]. Moreover, lower leg muscle [17] or thigh muscle volume [18] have been identified as preferable allometric scaling denominators to normalize VO_2peak_ compared with body mass or either fat-free mass (FFM).

The method for determining body composition should be selected depending on research objectives. In general, methods have been developed with specific assumptions [2, 8, 9, 19] which limits is use to a certain group; such as males or females, general population or athletes, adults or children. At the organ and tissue level, imaging techniques such as computed axial tomography (CT) and multi-scan magnetic resonance imaging (MRI) are considered the “criterion” methods in assessing body composition [20, 21]. The image quality of CT is improving [22] but, compared to exposures of 2.5 mSv of typical natural environments, the effective radiation exposure is still ~10 mSv for a full body scan [21]. MRI, although safe, requires a high technical proficiency and is not cost-effective. Dual-energy X-ray absorptiometry (DXA) uses a relatively low dose of ionizing radiation (i.e., 1 mSv per scan) and is considered the safest and appropriate imaging modality to accesses body composition [23]. Still, the selection also needs to consider practical implications such as cost, operation, required technical skills, subject cooperation. Accessible, safe and cost-effective strategies are needed.

Water displacement leg volumetry offers a simple approach and was used for validation of lower limb volume derived from six truncated cone formulas in 32 young male and 15 adult females [24]. Studies on the validation of this anthropometric protocol in athletes are still limited to male rugby players [25]. In addition, in a sample of male adult cadavers midthigh girth was the best predictor of skeletal mass with a coefficient of correlation of 0.94 [9]. Therefore, the current study examines the validity of thigh volume (TV) estimated by anthropometry relative to estimates by DXA as the reference method in late adolescent female volleyball players. The analysis also considered a calibration of the anthropometric approach proposed by Jones and Pearson [24] by adding supplemental variables.

## Methods

### Study design and participants

The current cross-sectional study was part of a research project conducted in the Midlands of Portugal. Participants were recruited voluntarily from four competitive clubs covering three urban areas (Aveiro, Coimbra and Leiria). All volleyball players were from middle and high socio-economic status; considering parents educational background [26]. Adolescents were students in elementary and secondary schools. A total of 54 female adolescent volleyball players were assessed. The following inclusion criteria were considered: (*i*) chronological age >14.0 years and <17.9 years; (*ii*) based on self-reported information reaching menarche > 1 year (range: 1.5–6.4 years) before testing; (*iii*) having all data completed. The final sample of the current study was composed of 42 female volleyball players aged 14.0–17.9 years of European (Caucasian) ancestry.

### Procedures

All research procedures were reviewed and approved by the Sports Science and Physical Education Faculty Scientific Committee of the University of Coimbra (CE/FCDEF-UC/00102014). Informed and formal written consent was acquired from all parents or legal guardians. During Easter breaks (i.e., 2-week period) of 2014 and 2015, coaches and athletes visited the laboratories in groups of 4–6 players during weekday afternoons. All measurements were taken in the same day. The anthropometric data was collected in standardized conditions by single and highly trained technician (MJCS), adopting recommended procedures [27]. DXA measurements were performed at the Department of Medical Imaging and Radiation Therapy at the School of Health and Technology of Coimbra. A single laboratory professional positioned the participants, performed the scans and executed the analysis according to the manufacturer’s manual.

### Training history

Volleyball training years were obtained by individual interview at clubs and confirmed through the records at the Portuguese Volleyball Federation. Annual training volume for each player was daily recorded by the head coaches using standardized reporting forms of the research project. Players had ~3 training sessions per week of ~90 min∙session^-1^ and usually one game on Saturdays. Injuries and illnesses were accounted as nonattendances to training in the final annual training volume calculations.

### Anthropometry

Stature (Harpenden stadiometer, model 98.603, HoltainLtd, Crosswell, UK), body mass (Seca electronic scale, model 770, Hamburg, Germany) and two skinfolds (Lange caliper, Beta Technology, Ann Arbor, MI, USA) were measured. Anterior and posterior skinfold measurement was performed on the right side of the body, to the nearest 0.1 mm, at the same level as mid-thigh circumference. The posterior thigh skinfold is not previewed in the above-cited standardized procedures but was already adopted in previous studies dealing with school children aged 10–13 years of both sexes [28, 29] and male adolescent rugby players [25]. Standing up, the participant places the right foot at the edge of the box with the knee bent at a right angle. The posterior skinfold site is then measured on the midline of the thigh with the technician kneeling and facing the left side of the participant.

### Non-invasive estimation of thigh volume

Thigh volume estimated by anthropometry (TV-ANTH) of the right leg was determined from three circumferences and two partial lengths measured two times each and averaged for analysis. The technique was based on the procedures proposed by Jones and Pearson [24] in which the TV is fractioned into two portions, like two truncated cones: truncated cone 1 was determined from circumferences measured at the most proximal gluteal furrow and at one-third of the subischial height up from the tibial–femoral joint space; truncated cone 2 was similarly determined by using circumferences at the one-third of the subischial height up from the tibial–femoral joint space and at the transversal plan above the patella. Further details are provided in a clinical study [30]. In brief, volume was estimated from an equation derived from proximal (*A*_1_) and distal (*A*_2_) area sections that composed the truncated cones, and *L* is the length between the two transverse plans:

1 $$ TV- ANTH=\left({\left[{A}_1+{A}_2+\left({A}_1\times {A}_2\right)\right]}^{0.5}\right)\times \frac{1}{3}\times L $$

The areas *A*_1_ and *A*_2_ were derived from leg circumferences:

2 $$ A = \frac{C^2}{4\times \pi } $$

### DXA

Whole-body DXA scans were performed with a Lunar DPX-PRO/NT/MD+ densitometer (Software Lunar Encore for Windows version 13.6, Waltham, MA, USA). Participants were positioned and examined according to the manufacturer’s manual. Regional composition of the lower limbs was sectioned following the same anatomic landmarks used in the anthropometric protocol. To derive thigh volume (TV-DXA), thigh lean soft tissue and bone were summed to obtain FFM in kilograms. Fat mass (FM) was also derived in kilograms. TV-DXA was calculated as:

3 $$ TV-DXA=\frac{{\mathrm{FFM}}_{\mathrm{DXA}}}{1.1}+\frac{{\mathrm{FM}}_{\mathrm{DXA}}}{0.9} $$

The 1.1 g · mL^-1^ and the 0.9 g · mL^-1^, represents the mean density for FFM and FM, respectively [31].

### Data quality

An independent sample of 20 late adolescent athletes from several sports was evaluated in all anthropometric dimensions two times within one week by the same technician (MJCS). Technical error of measurement (TEM; [32]) for stature and body mass were 0.32 cm and 0.21 kg, respectively. Corresponding values for front and posterior thigh skinfolds were 0.91 mm. For circumferences, TEM were 0.84 cm for proximal, 0.50 cm mid-thigh and 0.50 cm for distal. For the two partial lengths, TEM were 0.62 cm and 0.44 cm. The obtained errors of measurers are within the range of errors of a variety of field surveys [32]. Test-retest of DXA scans in 10 individuals displayed a coefficient of variation of 0.8% and 1.6% for lean soft tissue and percent of FM, respectively.

### Data analysis

Descriptive statistics for the total sample of female adolescent volleyball players were calculated and the Kolmogorov-Smirnov test was used to analyse the degree of normality. Log_10_-transformations were applied to reduce nonuniformity of errors. The interrelationship between body mass and estimates of TV (TV-DXA and TV-ANTH) were examined using Pearson Correlation. Years of training, annual number of training sessions, chronological age, age at menarche, log_10_ (stature), log_10_ (body mass), log_10_ (anterior thigh skinfold) and log_10_ (posterior thigh skinfold), were included in the stepwise regression model as potential independent variables with log_10_ (TV-DXA) as the dependent variable. To avoid collinearity a variance inflation factor < 10 and a tolerance > 0.10 was set [33]. Individual values of predicted TV were calculated by back-transformation of the values generated by the new model (TV-NM). The predicted residual sum of squares (PRESS) was used to validate the regression model [34]. Differences between estimated TV by the new predictive model and TV estimated by DXA were evaluated with paired-sample Student’s *t*-tests. Agreement between anthropometric estimates (practical approach) and volume based on DXA was assessed using Deming regressions [35]. This regression method was adopted since both *x* and *y* variables are subject to error. Limits of agreement between TV-NM and TV-DXA were assessed with Bland-Altman plots [36]. Alpha level was set at 0.05. Data analyses were completed using IBM SPSS v.23 for Mac OS.

## Results

Descriptives for training experience, chronological age, estimated age at menarche, stature, body mass and appendicular skinfolds are summarized in Table 1. Body mass and posterior thigh skinfold did not fit the assumptions of normal distribution; all non-normally distributed variables were log transformed. All volleyball players attained menarche (ranged 10.8–15.7 years) and compete in organized and competitive volleyball (2-8 years). Mean statures and body masses (±SD) were 165.2 ± 0.9 cm and 61.1 ± 1.4 kg, respectively.

**Table 1:** Descriptive statistics for the total sample of female adolescent volleyball players (*n* = 42) and test of normal distribution

	Range	Mean			SD	Kolmogorov-Smirnov	
		Value	95% CI	SE		Value	P-value
Training (years)	2-8	4.21	3.57-4.85	0.32	2.85	0.113	0.200
Training sessions (#)	30-129	92.5	85-100	3.7	24.1	0.132	0.062
CA (years)	14.00-18.76	16.76	16.40-17.12	0.18	1.16	0.091	0.200
Age at menarche (years)	10.75-15.67	12.72	12.44-13.00	0.14	0.90	0.097	0.200
CA – age at menarche (years)	1.46-6.39	4.04	3.67-4.41	0.18	4.41	0.088	0.200
Stature (cm)	154.8-175.6	165.2	163.5-166.9	0.9	5.5	0.093	0.200
Body mass (kg)	47.5-88.1	61.1	58.2-64.0	1.4	9.4	0.145	0.026
Skinfold thigh anterior (mm)	14-38	24.7	23.0-26.4	0.8	5.4	0.111	0.200
Skinfold thigh posterior (mm)	17-42	26.3	24.2-28.5	1.1	6.9	0.148	0.022

*CA* chronological age, *CI* confidence intervals for the mean, *SE* standard error of the mean, *SD* standard deviation

TV estimates derived from anthropometry and by DXA are summarized in Table 2. The means and standard deviation for TV-ANTH and TV-DXA were 4.91 ± 1.03 L and 5.55 ± 0.90 L, respectively. TV-ANTH did not fit the assumptions of normal distribution and were subsequently log-transformed.

**Table 2:** Thigh volume estimates by anthropometry and by dual X-ray absorptiometry in female adolescent volleyball players (*n* = 42) and test of normal distribution

	Range	Mean			SD	Kolmogorov-Smirnov	
		Value	95% CI	SE		Value	*P*-value
TV-ANTH (L)	3.67-8.49	4.91	4.59-5.23	0.16	1.03	0.157	0.011
TV-DXA (L)	4.17-8.00	5.55	5.27-5.83	0.14	0.90	0.123	0.113

*TV-ANTH* thigh volume estimated by anthropometry, *TV-DXA* thigh volume estimated by dual X-ray absorptiometry, *CI* confidence intervals for the mean, *SE* standard error of the mean, *SD* standard deviation

**Fig. 1:**
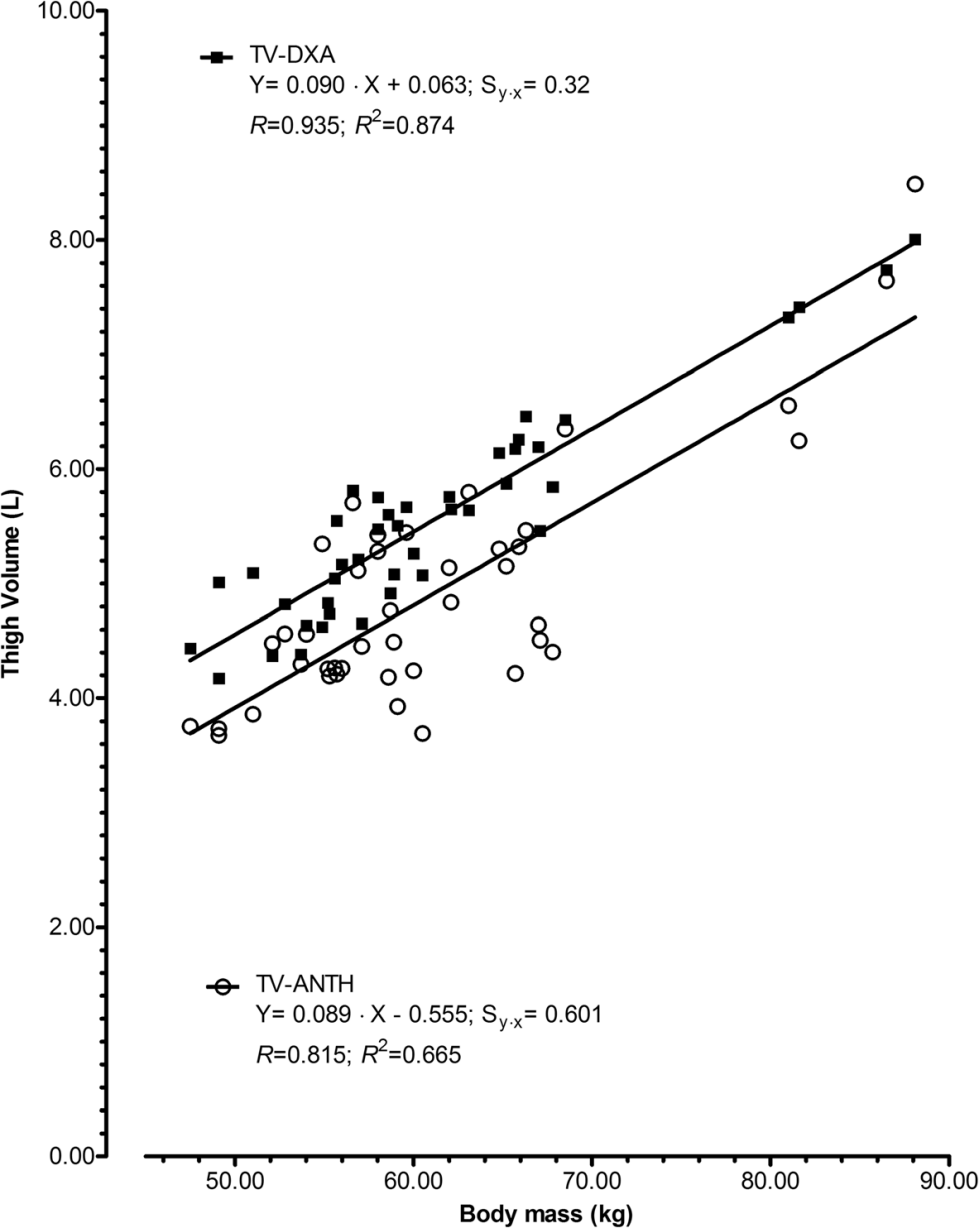
Regressions between body mass and estimates of thigh volume by anthropometry (TV-ANTH) and by dual X-ray absorptiometry (TV-DXA) in female volleyball players aged 14-18 years (*n* = 42). The standard error of estimation (Sy∙x), correlation (*R*) and coefficient of determination (*R* ^2^) are also presented

**Fig. 2:**
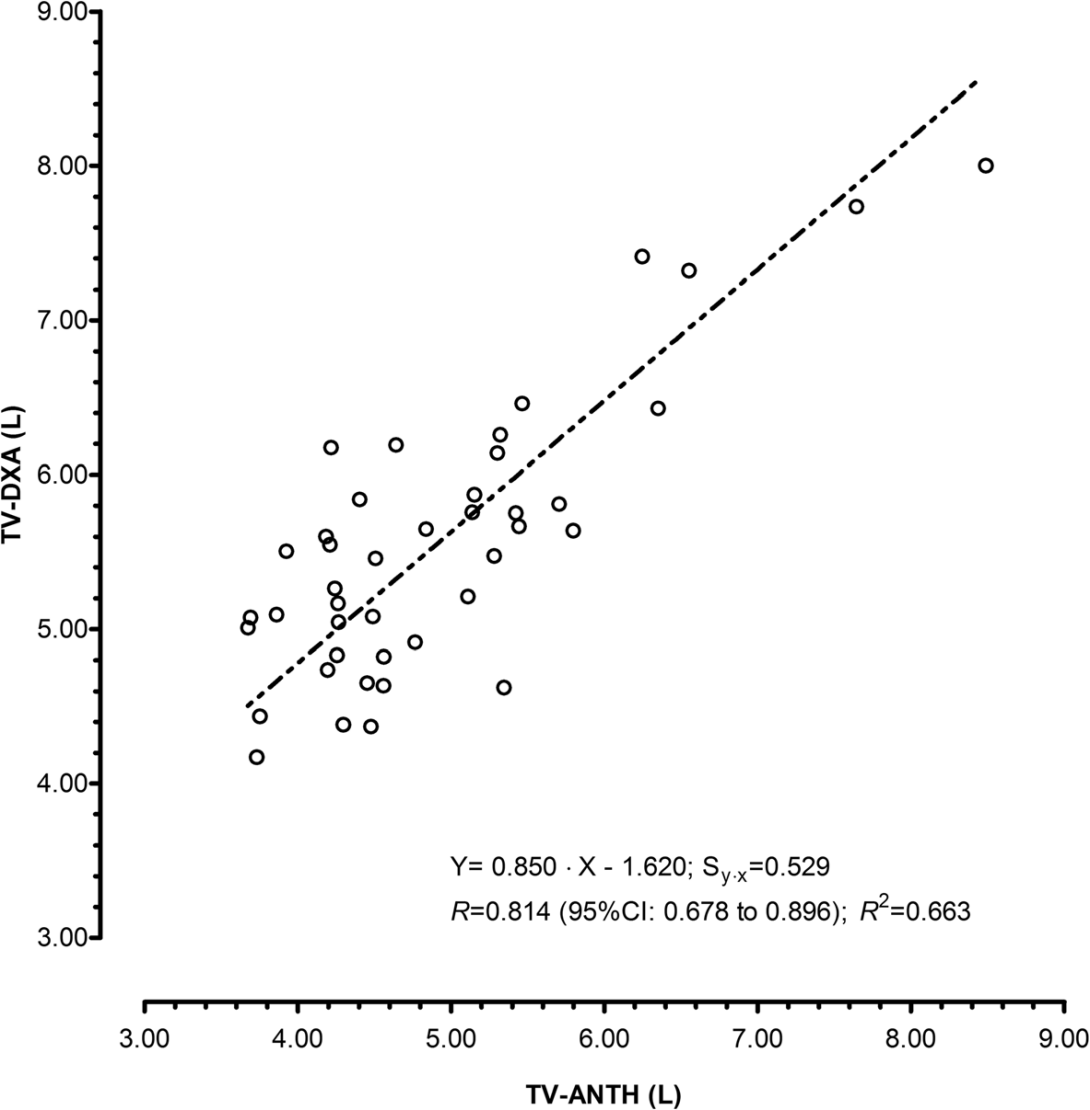
Deming regression between estimates of thigh volume by anthropometry (TV-ANTH) and by dual X-ray absorptiometry (TV-DXA) in female volleyball players aged 14-18 years (*n* = 42). The standard error of estimation (Sy∙x), correlation (*R*), confidence intervals for the correlation (CI) and coefficient of determination (*R* ^2^) are also presented

Relationships between body mass and estimates of TV (TV-ANTH; TV-DXA) are illustrated in Fig. 1. The correlation coefficients were 0.935 (95%CI: 0.882–0.965; 87.4% of variance in TV-DXA explained by body mass) and 0.815 (95%CI: 0.680–0.897; 66.5% of variance in TV-ANTH explained by body mass). The concurrent assessments of TV (TV-ANTH; TV-DXA) presented a large correlation (0.814; 95%CI: 0.678–0.896) with 66.37% of overlapping variance (Fig. 2). The slope coefficient was 0.850 (95%CI: 0.656–1.04) and was not considered different from the unit. The intercept was 1.620 (0.408–2.350) and was not significantly different from zero. A slope coefficient different from one reflects a proportional error while a systematic error corresponds to an intercept different from zero [35, 37, 38].

The regression model for TV prediction is given in Table 3. The correlation between the TV-NM including log_10_ (body mass), log_10_ (TV-ANTH) with TV-DXA was 0.922 (85.1% of overlapping variance). The prediction equation was as follows:

**Table 3:** Regression model to obtain log-transformed estimates of thigh volume in female adolescent volleyball players and internal cross-validation analysis for the new model using PRESS

X_i_	Intercept and predictors						Model summary				PRESS	
	*β*	SEE	P-value	Partial-*r*	VIF	1/VIF	*R*	SEE	*R* ^2^	*R*_adj_^2^	*R* ^2^	SEE
Intercept	-0.899	0.153	<0.001				0.922	0.027	0.851	0.843	0.838	0.027
log_10_ (body mass)	0.876	0.108	<0.001	0.810								
log_10_ (TV-ANTH)	0.113	0.081	0.172	0.138	0.386	2.589						

*TV-ANTH* thigh volume estimated by anthropometry, *β*, beta unstandardized, *SEE* standard error or estimate, *r* and *R*: correlation, *VIF* variance inflation factor, *1/VIF* tolerance, *R* ^2^ coefficient of determination, *R*_adj_^2^: adjusted coefficient of determination

4 $$ \mathrm{T}\mathrm{V}-\mathrm{N}\mathrm{M}\kern0.5em =-0.899+0.876 \times { \log}_{10}\ \left(\mathrm{body}\ \mathrm{mass}\right)+0.113 \times \kern0.5em { \log}_{10}\kern0.75em \left(\mathrm{T}\mathrm{V}-\mathrm{ANTH}\right) $$

PRESS statistics were used to internally validate the TV-NM as presented in Table 3. The *R* ^2^ (0.838) was higher than *R* ^2^ presented in Fig. 2 (0.663) and SEE in Table 3 was low (0.027) which emphasized the accuracy of the model.

**Fig. 3:**
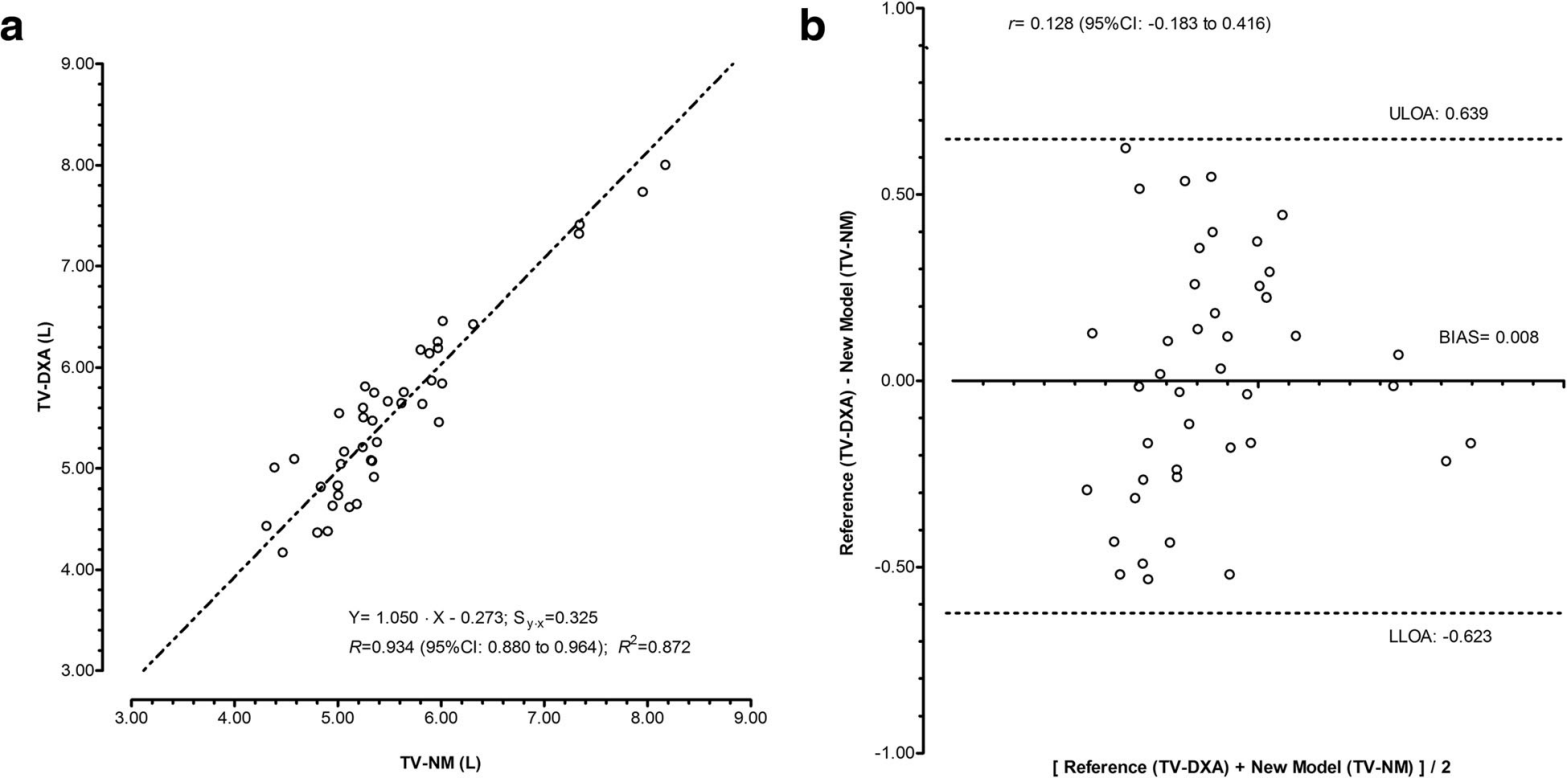
Linear least product regressions between estimates of thigh volume and relationship between residuals and the mean of the concurrent estimates in female volleyball players aged 14-18 years (*n* = 42). Estimates of thigh volume by anthropometry-based new model (TV-NM) and by dual X-ray absorptiometry (TV-DXA, panel **a**). Differences between anthropometry-based new model (TV-NM) and reference given as dual X-ray absorptiometry (TV-DXA, panel **b**). The standard error of estimation (Sy∙x), correlation (*R* and *r*), coefficient of determination (*R* ^2^), upper limits of agreement (ULOA) and lower limits of agreement (LLOA) are also presented

Deming repression analysis determined a nearly perfect correlation between TV-DXA and the TV-NM proposed in the current study for female adolescent volleyball: *R* = 0.934 and a Sy∙x = 0.325 (Fig. 3a). As reported in Fig. 3a, the intercept (-0.273) and slope (1.050) of the equation did not significantly differ from zero and one, respectively, rejecting the possibility of systematic or proportional bias. The measurement differences (error) relative to magnitude of TV were homoscedastic (r = 0.128; 95%CI: -0.183 to +0.416; Fig. 3b) with no significant mean differences between TV estimates by the ANTH-based NM and by DXA (Table 4).

**Table 4:** Thigh volume estimates by the anthropometry-based new model and by dual energy X-ray absorptiometry in female adolescent volleyball players, mean differences between methods and paired *t*-test (*n* = 42)

	Methods				Differences			Paired *t*-test	
	TV-DXA		TV-NM						
	Mean	SD	Mean	SD	Mean	95%CI	SD	t_(df=41)_	P-value
Thigh volume (L)	5.545	0.858	5.553	0.899	-0.899	-0.108–0.092	0.322	0.160	0.873

*TV-DXA* thigh volume estimated by dual X-ray absorptiometry, *TV-NM* thigh volume estimated by anthropometry-based new model, *SD* standard deviation, *CI* confidence intervals for the mean differences

## Discussion

The present study developed and cross-validated an anthropometric equation for estimating TV based on DXA assessments in female late adolescent athletes. Estimated TV using the anthropometric equation [24] with adjustments for body mass among predictors increased the precision of the estimates relative to assessments by DXA which is considered a non-invasive imaging and reference technique. The data of the present study suggested that TV tended to be underestimated when derived from anthropometric models that combine the sum of two truncated cones compared to measurements derived from DXA assessments, although concurrent estimates were highly correlated (*R* = 0.814; 95%CI: 0.678–0.896). Among the limitations of this studies is the fact that DXA assumes constant density for all its components (i.e., skeletal mass tissue = 1.04 g · mL^-1^) and small errors into DXA estimates of skeletal mass may be introduced by the variability in actual skeletal mass density [12, 39]. Also, for the conversion of values of mass to volume, constant densities of FFM and FM values are used. It is known that actual densities may vary between different genders, ethnic groups and according to age [40].

Anthropometric equations for the prediction of total body lean soft tissue were developed in a large sample of nonobese and obese adults using MRI as the reference method [13]. These models are based in several assumptions: (*i*) lean soft tissue is theoretically in the shape of a cylinder; (*ii*) skinfolds can be used to correct measures of limb circumferences and provide reliable estimates of limb lean soft tissue circumferences; and, (*iii*) that the squared value of appendicular limb lean soft tissue circumferences generate estimates of lean soft tissue area. Though the accuracy of these models in predicting skeletal mass in adults exists [10], evidence of its applicability in young athletes is still lacking. In the current study, the difference between total body mass derived from scale mass and DXA was less than 1%. This value is within the range of under- or overestimation of whole-body FM using DXA due to extracellular fluid variations [41]. DXA-determined appendicular lean soft tissue has been validated against CT [31, 42]. This method was proposed as a reference imaging technique for the evaluation of limb lean soft tissue [10–13]. The method enables the identification of particular regions of interest and separates respective masses into bone mineral content plus two tissue compartments: fat and lean soft tissue [43].

The ordinary least squares method is generally used to obtain the slope and intercept of linearly related data. The technique presumes that the comparative method values are without error [44]. However, when random error affects both *y* and *x* values the least squares method may not be appropriate. Deming regression is a promising alternative because accommodates both *y* and *x* errors [25, 29, 45].

The sample of the current study was smaller than the 268 participants used in a previous study [6] but comparable with the study of rugby players aged 19.9 ± 2.2 years (*n* = 41) [25]. An appropriate participant-to-variable ratio is considered a relevant aspect while using multiple regressions, and it is recommended to maintain the critical proportion higher than 5:1 and preferably 20:1 [46]. In the current study, the baseline ratio was 5:1 with eight candidate predictors. The final model was based on a ratio of 21:1 with two significant predictors obtained from a sample of 42 female volleyball players. The PRESS method was used to avoid data splitting [34] which is a well-known method where the complete sample data are typically split into a fitting sample and a validation sample [37, 38]. The term PRESS corresponds to an acronym for predicted residual sum of squares. After obtaining a candidate model using multiple linear regressions analysis, the PRESS technique involves different prediction equations to obtain independent residuals since each case is left out of the respective model for estimation purposes.

A previous study with Portuguese adolescents athletes [6] was the first to present anthropometric-based equations to estimate limbs lean soft tissue with DXA as the reference method. Body mass was the strongest predictor of DXA-measured appendicular lean soft tissue, explaining 83% of the variation in appendicular lean soft tissue, while sex and stature contributed with 5% and 3% of additional explained variance. Chronological age only added 0.4% of the explained inter-individual variability. Among 65 boys and girls below stage 5 of sexual maturation, it was demonstrated that the stages of sexual maturation failed to contribute in the prediction of skeletal muscle after including body weight [11]. However, it should be noted that sexual maturation was determined by combining self-assessed stages of breast and pubic hair development, which could lead to misclassification between genders [40]. It is believed that appendicular fat tissue and appendicular lean soft tissue may be differently affected by inter-individual variability of biological maturation [47], since, on average, boys linearly increase muscle mass whereas girls attain a plateau in muscle mass and accumulate larger amounts of fat mass, particularly in the extremities. A recent study in healthy circumpubertal boys [29], used predicted mature (adult) stature as a non-invasive maturity status and identified a positive and significant contribution of this variable to explain inter-individual differences in lower limbs lean soft tissue. Additional studies are necessary to check for the adequacy of sex-specific models, while also using a valid assessment of biological maturity.

The allometric modelling procedures of the present study did not identified a significant contribution of years of training, annual number training sessions, chronological age, age at menarche, stature or posterior thigh skinfold to predict TV. Years of training are strongly correlated with chronological age and both variables presented a small range of variability and normal distribution. On the other hand, annual number of training sessions does not represent specify and intensity of the training activities. Similarly to previous studies [6, 25, 28, 29], body mass (*R* = 0.810) explained most of the variance of the TV-NM proposed in the current study for female adolescent volleyball players. The estimated TV using the anthropometric equation [24] added little explained variance (*R* = 0.138). Other approaches to estimate appendicular volume without the inclusion of TV-ANTH should be explored.

The available literature was lacking in non-invasive models to estimate appendicular size descriptors for female adolescent athletes. Estimates of lower limbs soft tissue or volume [16–18] are highly and positively associated with a multiplicity of ergometer and fitness assessments. Also, they have been shown to be more suitable scaling denominators than body mass or FFM to predict peak oxygen uptake in adolescent (14.5–16.5 years) male roller hockey players [16] and to predict outputs resulting from short-term power assessments in adolescent (14.0–16.0 years) male basketball players [5]. The equation derived from the present study is a useful analytical tool for the critical interpretation of variation of TV within and between individuals and presented similar internal validity to other available studies for similar proposes [6, 25, 28, 29].

It was possible to obtain an acceptable and nonbiased method to accurately predict thigh volume in female volleyball players aged 14–18 years. Strengths of the present study include the analysis of agreement between thigh volume predicted by a new anthropometry based model and estimated with DXA; which indicated no proportional bias between methods (see Fig. 2). Also, conclusions of the present study cumulatively emerged from the PRESS statistics combined with the analysis of mean differences and also from the plot of individual differences by mean scores of the measurements defining upper and lower limits of agreement. A substantial percentage of variance was explained by the new predictive model (*R* ^2^ = 0.872) and body mass was a relevant and additional predictor. The current investigation is not without limitations. The study was limited to a relatively small sample of 42 female volleyball players from a narrow age range. Moreover, prediction equations tend to be population specific and additional validation studies in female adolescent volleyball players are required to validate this model.

## Conclusions

The current study clearly identified a new anthropometric-based model to estimate TV in research dealing with female late adolescent athletes. The model is based on procedures proposed by Jones and Pearson [24] with the addition of simple body mass measures. It was internally cross-validated with suitable statistical procedures, ensuring its applicability to similar samples. This equation represents an accurate and practical alternative to quantify TV in late adolescent female volleyball players.

## Additional file

Additional file 1: Dataset used in this Research. YT: years of training; ANS: annual number of training sessions; ANG: annual number of games; BM: body mass; H: stature; SKTA: anterior mid-thigh skinfold; SKTP: posterior mid-thigh skinfold; PTL: proximal thigh length; DTL: distal thigh length; TTL: total thigh length; CGF: circumference at the most proximal gluteal furrow (highest possible horizontal circumference); CMT: circumference at the one-third of the subischial height up from the tibial–femoral joint space; CMTC: circumference at the one-third of the subischial height up from the tibial–femoral joint space corrected for subcutaneous adipose tissue thickness; CDT: circumference at the minimum circumference above the patella (distal thigh); TVANTH: thigh volume based on Jones and Pearson; TVANTHFF: fat free volume in the thigh calculated based on Jones and Pearson; TVANTHF: fat volume in the thigh based on Jones and Pearson; DXATV: thigh volume estimated by DXA; DXATVFF: fat free volume in the thigh estimated by DXA; DXATVF: fat volume in the thigh estimated by DXA. (XLSX 21 kb)

## Abbreviations

A: Area sections
C: Circumferences
CT: Computed axial tomography
DXA: Dual-energy X-ray absorptiometry
FFM: Fat-free mass
FM: Fat mass
MRI: Magnetic resonance imaging
PRESS: Predicted residual sum of squares
TEM: Technical errors of measurement
TV: Thigh volume
TV-ANTH: Thigh volume estimated by anthropometry
TV-DXA: Thigh volume estimated by DXA
TV-NM: Thigh volume estimated by anthropometry-based new model
