# Erratum to: Concurrent agreement between an anthropometric model to predict thigh volume and dual-energy X-Ray absorptiometry assessment in female volleyball players aged 14-18 years

**DOI:** 10.1186/s12887-017-0818-8

**Published:** 2017-02-24

**Authors:** Óscar M. Tavares, João Valente-dos-Santos, João P. Duarte, Susana C. Póvoas, Luís A. Gobbo, Rômulo A. Fernandes, Daniel A. Marinho, José M. Casanova, Lauren B. Sherar, Daniel Courteix, Manuel J. Coelho-e-Silva

**Affiliations:** 1 UID/DTP/03213/2016, Faculty of Sport Sciences and Physical Education, University of Coimbra, Pavilhao III, 3040-156 Coimbra, Portugal; 2 Department of Medical Imaging and Radiation Therapy, School of Health and Technology, Instituto Politécnico de Coimbra, Coimbra, Portugal; 3 Faculty of Physical Education and Sport, Lusófona University of Humanities and Technologies, Lisbon, Portugal; 4 Portuguese Foundation for Science and Technology (SFRH/BPD/100470/2014), Lisbon, Portugal; 5 Research Center in Sports Sciences, Health Sciences and Human Development, CIDESD, University Institute of Maia, ISMAI, Maia, Portugal; 6 Laboratory of Investigation in Exercise (LIVE), Department of Physical Education, São Paulo State University (UNESP), São Paulo Presidente Prudente, Brazil; 7 Department of Sport Sciences, University of Beira Interior, Covilhã, Portugal; 8 Faculty of Medicine, University of Coimbra, Coimbra, Portugal; 9 School of Sport, Exercise and Health Sciences, Loughborough University, Loughborough, UK; 10 Laboratory of Metabolic Adaptations to Exercise in Physiological and Pathological Conditions, Clermont Auvergne University, Blaise Pascal University, Clermont-Ferrand, France; 11 School of Exercise Science, Faculty of Health, Australian Catholic University, Melbourne, VIC Australia; 12 Research Centre in Human Nutrition, Auvergne Clermont-Ferrand, France

## Erratum

Following the publication of this article [1] it was brought to our attention that the affiliation for author Susana C. Póvoas is incorrect and for author Daniel A. Marinho incomplete.

Susana C. Póvoas is erroneously affiliated with the Research Centre in Sport and Physical Activity, Maia Institute of Higher Education, Maia, Portugal. The author’s correct affiliation is with the Research Center in Sports Sciences, Health Sciences and Human Development, CIDESD, University Institute of Maia, ISMAI, Maia, Portugal.

Daniel A. Marinho, affiliated only with the Department of Sport Sciences, University of Beira Interior, Covilhã, Portugal in the original article [1], is also affiliated with the Research Center in Sports Sciences, Health Sciences and Human Development, CIDESD, University Institute of Maia, ISMAI, Maia, Portugal.

Both authors’ affiliations are correctly included in the Author details of this erratum.
